# Genome-Wide Association Mapping for Identification of Quantitative Trait Loci for Rectal Temperature during Heat Stress in Holstein Cattle

**DOI:** 10.1371/journal.pone.0069202

**Published:** 2013-07-23

**Authors:** Serdal Dikmen, John B. Cole, Daniel J. Null, Peter J. Hansen

**Affiliations:** 1 Department of Animal Science, Faculty of Veterinary Medicine, Uludag University, Bursa, Turkey; 2 Animal Improvement Programs Laboratory Agricultural Research Service, USDA, Beltsville, Maryland, United States of America; 3 Department of Animal Sciences, D.H. Barron Reproductive and Perinatal Biology Research Program, and Genetics Institute, University of Florida, Gainesville, Florida, United States of America; University of Queensland, Australia

## Abstract

Heat stress compromises production, fertility, and health of dairy cattle. One mitigation strategy is to select individuals that are genetically resistant to heat stress. Most of the negative effects of heat stress on animal performance are a consequence of either physiological adaptations to regulate body temperature or adverse consequences of failure to regulate body temperature. Thus, selection for regulation of body temperature during heat stress could increase thermotolerance. The objective was to perform a genome-wide association study (GWAS) for rectal temperature (RT) during heat stress in lactating Holstein cows and identify SNPs associated with genes that have large effects on RT. Records on afternoon RT where the temperature-humidity index was ≥78.2 were obtained from 4,447 cows sired by 220 bulls, resulting in 1,440 useable genotypes from the Illumina BovineSNP50 BeadChip with 39,759 SNP. For GWAS, 2, 3, 4, 5, and 10 adjacent SNP were averaged to identify consensus genomic regions associated with RT. The largest proportion of SNP variance (0.07 to 0.44%) was explained by markers flanking the region between 28,877,547 and 28,907,154 bp on *Bos taurus* autosome (BTA) 24. That region is flanked by *U1* (28,822,883 to 28,823,043) and *NCAD* (28,992,666 to 29,241,119). In addition, the SNP at 58,500,249 bp on BTA 16 explained 0.08% and 0.11% of the SNP variance for 2- and 3-SNP analyses, respectively. That contig includes *SNORA19*, *RFWD2* and *SCARNA3*. Other SNPs associated with RT were located on BTA 16 (close to *CEP170* and *PLD5),* BTA 5 (near *SLCO1C1* and *PDE3A*), BTA 4 (near *KBTBD2* and *LSM5*), and BTA 26 (located in *GOT1*, a gene implicated in protection from cellular stress). In conclusion, there are QTL for RT in heat-stressed dairy cattle. These SNPs could prove useful in genetic selection and for identification of genes involved in physiological responses to heat stress.

## Introduction

Heat stress in dairy cattle can compromise a variety of physiological functions including milk yield [1], reproduction [2] and immune function [3]. One strategy for reducing the magnitude of heat stress is to select animals that are genetically resistant to heat stress. Thermotolerant strains or breeds have been developed in many species of livestock [4]. Most of the negative effects of heat stress on animal performance are a consequence of either the physiological adaptations that homeotherms undergo to regulate body temperature or the adverse consequences of failure to regulate body temperature [4]. Thus, selection for regulation of body temperature during heat stress could result in animals that are resistant to deleterious effects of heat stress. In fact, two specific genes associated with effects of heat stress on milk yield, the slick gene [5] and an allele of *ATP1A1* [6], are also associated with lower rectal temperature (RT).

Core body temperature during heat stress is a heritable trait in dairy cattle, with early estimates varying from 0.15 to 0.31 [7] and a more recent estimate indicating a value of 0.17 [8]. Reliability of genetic estimates for rectal temperature, such as for other genetically-controlled traits [9], [10], should be improved by genome wide association studies (GWAS) to identify SNPs associated with regulation of rectal temperature. Quantitative trait loci (QTL) can be identified for lowly-heritability traits and used to improve reliability of genetic estimates despite the gain in reliability being less than for more heritable traits [11], [12]. In addition, GWAS can be useful for understanding the underlying biology of a trait by identifying candidate genes in physical proximity to QTL [11], [13], [14].

The objective of the current study was to perform a GWAS for rectal temperature during heat stress in lactating Holstein cows and identify SNPs that serve as QTL for that trait.

## Materials and Methods

Use of animals was approved by the University of Florida Institutional Animal Care and Use Committee (Approval No. 2009-03578).

### Data Collection

The study was conducted with lactating Holstein cows located in Florida dairies at nine locations in Bell (29.75′° N, 82.86° W), Hague (29.77° N, 82.42° W), Okeechobee (27.24° N, 80.83° W) and Trenton (29.61° N, 82.82°W). The following dairies allowed access to cows and records: Alliance Dairy (Trenton, Florida), Hilltop Dairy (Trenton, Florida), Larson Dairy (Okeechobee, Florida; three locations), McArthur Dairy (Okeechobee, Florida; two locations), North Florida Holsteins (Bell, Florida) and the University of Florida Dairy Unit (Hague, Florida). All except the University of Florida Dairy Unit are private. For seven locations, cows were housed in free-stall barns equipped with fans and sprinklers. In the other two locations, some cows were housed in free-stall barns and other cows were housed in tunnel-ventilation barns. Cows were fed total mixed rations. Data on RT were collected from a total of 11,128 cows. A subset of these data have been analyzed previously [8], [12].

Rectal temperature was measured between 1500 and 1700 h during the months of June to September in 2007 and between 1400 and 1600 h in the months of June to September in 2010, 2011 and 2012. Each cow was measured once during the experiment. Rectal temperature was measured using a digital GLA M750 thermometer (GLA Agricultural Electronics, San Luis Obispo, CA, USA). Measurements were recorded while cows were under shade and, generally, while in head locks (for free-stall barns) or while resting (for tunnel-ventilation barns).

Measurements of dry bulb temperature (T_db_), relative humidity (RH), dew point temperature (T_dp_) and black globe temperature (T_bg_) inside the barn and T_bg_ outside the barn were measured at 1 min intervals during the period of data collection using a HOBO-U12 data logger (T_db_, RH, and T_dp_) and HOBO Water Temp Pro V2 data loggers (T_bg_) (Onset Company, Bourne, MA, USA). Measurements were taken at a position that was 2 m from the ground at a position in the center of the barn where cows were housed. Rectal temperature was matched with measurements of T_db,_ RH, T_dp_, and T_bg_ to the nearest minute at which environmental variables were recorded. Temperature humidity index (THI) [15] was calculated as follows:
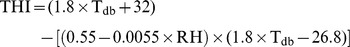
where T_db_ = dry bulb temperature (°C), and RH = relative humidity (%).

Data collected from days in which the THI at the time of collection was ≥78.2 were retained for genomic analysis. This THI was chosen because it is an indication that heat stress was sufficient to cause hyperthermia [16]. After restricting records to those cows undergoing heat stress and requiring that cows have known sire and dam, records were available from 4,447 cows sired by 220 bulls. The associated pedigree files included 12,346 animals. Of these phenotyped animals, 1,451 animals (107 cows and 1,344 bulls) had available genotypes using the Illumina BovineSNP50 BeadChip (Illumina, Inc., San Diego, CA, USA). Animals and SNP with call rates <0.90, SNP with minor allele frequencies <0.05, monomorphic SNP, and animals with parent-progeny conflicts were dropped from the dataset as part of the quality control process, leaving genotypes for 39,759 SNP from 1,440 individuals. A pedigree file including 12,346 male and female ancestors traced back to 1960 was obtained from the National Dairy Database maintained by the Animal Improvement Programs Laboratory (Beltsville, MD, USA).

Rectal temperatures were matched with performance data from the test-day closest to the date on which RT measurements were taken. Summary statistics for daily milk yield on the days when RT was measured, parity, DIM, and environmental variables are shown in Table 1.

**Table 1:** Average (±SE) and range of animal and environmental variables used in the analysis.^a^

	Mean (±SE)	Range (min. – max.)
Rectal temperature (°C)	38.8±0.01	37.2–41.6
Daily milk yield (kg)	34.1±0.13	0.9–74.1
Parity	2.0±0.02	1–8
DIM, (days)	185±1.48	1–996
T_db_ (°C)	31.7±0.03	26.3–41.7
RH (%)	64.3±0.18	32.5–92.6
T_dp_ (°C)	23.8±0.03	15.0–30.9
T_bg_ (°C), outside	36.1±0.07	22.7–48.1

### (Co)variance Components

(Co)variance components were estimated by restricted maximum likelihood [17] using the REMLF90 program [18]. Due to the limited number of observations available, genomic data were included for (co)variance components estimation. The estimates of Dikmen et al. [8] were used as starting values, and convergence was declared when the change between successive rounds of iteration was <10^−6^.

### Single-Step Genomic BLUP

A single-step genomic BLUP (ssGBLUP) method that integrates phenotypes, genotypes, and pedigree information was used to simultaneously estimate genomic predicted transmitting ability (PTA) and allele substitution effects [19]. Single-step methods provide greater power for QTL detection and more precise estimates of breeding values than two-stage BLUP by using a properly scaled and augmented relationship matrix [20], [21].

The single-trait model used for ssGBLUP analysis of RT was
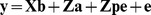
where **y** is a vector of RT phenotypes, **b** is a vector of fixed effects, **a** is a vector of additive genetic effects, **pe** is a vector of permanent environmental effects, and **e** is a vector of random residual effects. Fixed effects included parity, year of data collection, stage of lactation (separated into three stage of lactation classes: DIM <100; DIM between 100 and 200; and DIM >200), location of cows at measurement (resting or in headlocks), farm type (tunnel ventilation and regular free-stall barn), technician who measured RT, and farm. THI and milk yield at the time of measurement were included as covariates. **X_b_**, **Z_a_**, and **Z_pe_** are incidence matrices relating a record to fixed environmental effects in **b** and to random animal and permanent environmental effects in **a** and **pe**, respectively. Results were calculated using a Bayes A model as implemented in the BLUPF90 program [18] modified for genomic analyses [22]. Genome-wide association analyses for RT were conducted with individual SNP effects, as well as moving averages of 2, 3, 4, 5, and 10 consecutive SNP, using the POSTGSF90 package (I. Aguilar, INIA, Las Piedras, Canelones, Uruguay, 2012, personal communication).

Manhattan plots were created using ggplot2 0.9.2 [23] and R 2.15.1 [24] on an IBM xSeries 3850 server (IBM Corporation, Armonk, NY, USA) running Red Hat Enterprise Linux 5.0 (Red Hat, Inc., Raleigh, NC). Genomic calculations also were performed on that server.

## Results and Discussion

### Identification of QTL Associated with RT

Wang et al. [19] found in a simulation study that the correlation of QTL with the sum of adjacent SNPs increased up to 8 SNP, and then decreased as the number of summed SNP increased. This is because the closest SNP to a QTL is not always the best predictor of the QTL effect [25]. In the current study, 2, 3, 4, 5, and 10 adjacent SNP were averaged in order to identify consensus genomic regions associated with RT. A GWAS based on ssGBLUP was used in place of more traditional GWAS approaches because the latter do not directly use phenotypes of non-genotyped individuals. P-values were not used to declare regions as significant because such values are difficult to define and compare using classical hypothesis tests when shrunken estimators such as PTA are used [26]. Recent studies also have found that traditional GWAS methods often produce large numbers (e.g., hundreds) of significant effects, most of which cannot be validated in follow-up studies [27]. The principal objective of this study was to identify genomic regions associated with regulation of RT, and this is not dependent on tests of statistical significance that are more appropriate for candidate gene-based studies.

**Figure 1:**
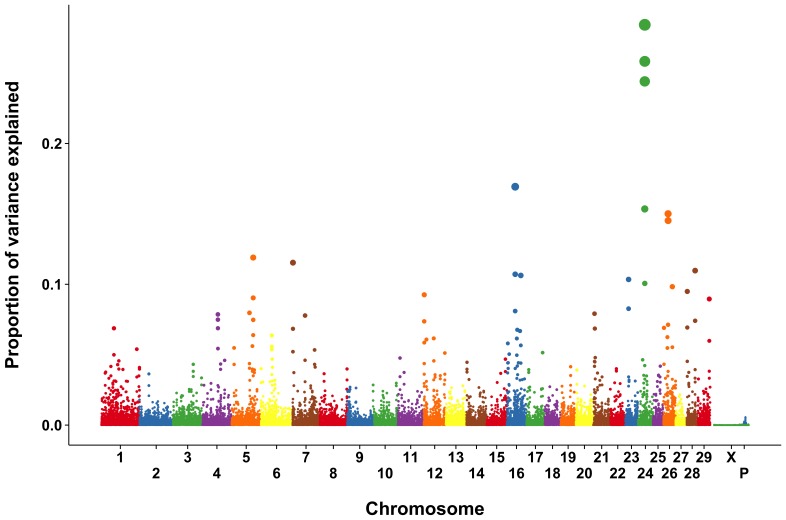
Proportion of SNP variance explained by 3-SNP moving windows for rectal temperature from a single-step GBLUP analysis.

**Figure 2:**
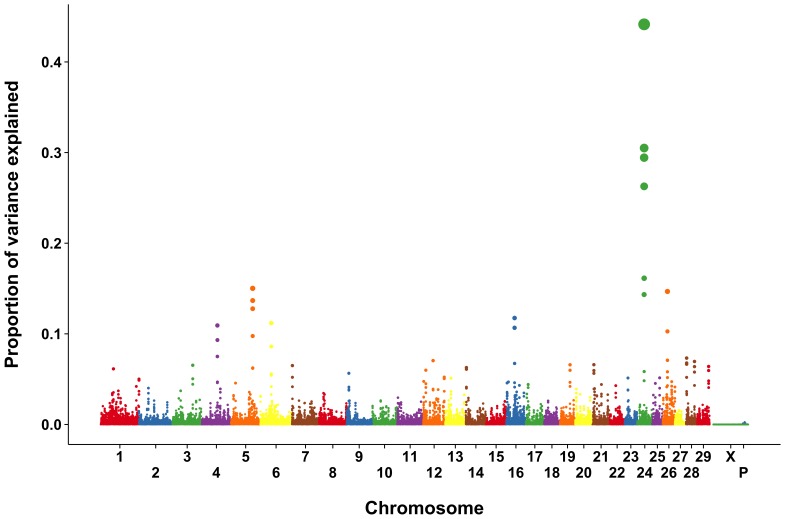
Proportion of SNP variance explained by 5-SNP moving windows for rectal temperature from a single-step GBLUP analysis.

The proportion of SNP variance explained by each marker individually is shown in Figure S1 and File S1. The individual SNP results were very noisy, and only one region on BTA 26 showed a clear signal. The use of 2-SNP windows (Figure S2) resulted in smoother marker effects, but it was still difficult to differentiate between markers with large effects and those with small effects. The Manhattan plots from the 3- and 5-SNP windows (Figures 1 and 2) resulted in clearer, and generally consistent patterns. Results were similar for the 4-SNP windows (Figure S3).

The 10-SNP results (Figure S4) are similar to the results found using narrower windows. Nonetheless the 10-SNP analysis poses problems because the Illumina BovineSNP50 BeadChip has an average intermarker spacing of 49.4 kbp so that each point on the resulting Manhattan plot covers an average of 494 kbp. This is problematic because useful linkage dsequilibrium (LD) in the cow extends for less than 100 kb [28]. Genes or groups of genes that are not in LD with one another but which have large effects should be represented by separate peaks.

The 20 largest explanatory loci for RT for each of the analyses is listed in Tables 2 and 3 and S1 through S4. Most commonly, the largest proportion of variance was explained by markers flanking the region between 28,877,547 and 28,907,154 bp on *Bos taurus* autosome (BTA) 24. That region is flanked by a U1 spliceosomal RNA (*U1*) on the left (28,822,883 to 28,823,043 bp) and a cadherin-2 (*NCAD*) gene on the right (28,992,666 to 29,241,119 bp). The U1 small ribonucleoprotein is involved in postranscriptional modification and regulation of mRNA length [29], both of which could be related to changes in gene expression in cells exposed to elevated temperature [30], [31]. *NCAD* was more highly expressed in paratuberculosis-infected cattle than in noninfected animals [32], but it is not clear what relationship, if any, there is between *NCAD* and stress responses.

**Table 2:** The 20 loci with the largest proportion of SNP variance explained for rectal temperature using 3-SNP sliding windows.

SNP name	Chromosome	Location (bp)	Variance explained (%)
BTB-01646599	24	28941584	0.28
ARS-BFGL-NGS-41140	24	28975828	0.26
Hapmap58887-rs29013502	24	28907154	0.24
BTB-00638221	16	35272426	0.17
BTB-01485274	24	28877547	0.15
ARS-BFGL-NGS-71584	26	20290497	0.15
ARS-BFGL-NGS-23064	26	20365711	0.15
BTB-01267098	5	89545151	0.12
ARS-BFGL-NGS-89847	7	2457750	0.12
BTA-26221-no-rs	28	35345760	0.11
Hapmap46698-BTA-38760	16	35317388	0.11
ARS-BFGL-NGS-108847	16	58500249	0.11
ARS-BFGL-NGS-29516	23	14246801	0.10
ARS-BFGL-NGS-35716	24	29013292	0.10
ARS-BFGL-NGS-95833	26	37797893	0.10
ARS-BFGL-NGS-16848	28	2924302	0.09
BTA-27496-no-rs	12	2500836	0.09
BTB-01267080	5	89512928	0.09
ARS-BFGL-NGS-107395	29	47527067	0.09
ARS-BFGL-NGS-100006	23	14215024	0.08

**Table 3:** The 20 loci with the largest proportion of SNP variance explained for rectal temperature using 5-SNP sliding windows.

SNP name	Chromosome	Location (bp)	Variance explained (%)
Hapmap58887-rs29013502	24	28907154	0.44
BTB-01646599	24	28941584	0.31
BTB-01485274	24	28877547	0.29
ARS-BFGL-NGS-41140	24	28975828	0.26
ARS-BFGL-NGS-35716	24	29013292	0.16
BTB-01267080	5	89512928	0.15
ARS-BFGL-NGS-10307	26	20259486	0.15
Hapmap54981-rs29019846	24	28853585	0.14
BTB-01267098	5	89545151	0.14
Hapmap47861-BTA-120563	5	89472174	0.13
ARS-BFGL-NGS-100932	16	35230105	0.12
Hapmap47403-BTA-76048	6	45153190	0.11
Hapmap39941-BTA-70878	4	64386271	0.11
ARS-BFGL-NGS-106628	16	35172012	0.11
BTB-00638221	16	35272426	0.11
ARS-BFGL-NGS-71584	26	20290497	0.10
BTB-01267042	5	89568937	0.10
ARS-BFGL-NGS-68143	4	64492908	0.09
Hapmap30420-BTC-039335	6	45175137	0.09
ARS-BFGL-NGS-458	4	64351574	0.08

Two regions of interest were flagged on BTA 16. The 2- and 3-SNP analyses (Table S2 and 2) found that the SNP at 58,500,249 bp explained 0.08 and 0.11% of the SNP variance, respectively. That contig includes a small nucleolar RNA (*SNORA19*) from 58,520,021 to 58,520,149 bp; a ubiquitin-protein ligase (*RFWD2*) from 58,600,678 to 58,838,844 bp; and small Cajal body specific RNA 3 (*SCARNA3*) from 58,628,128 to 58,628,268 bp. Small nucleolar RNA like *SNORA19* identify sites of pseudouridine formation [33], which may be involved in the initiation of translation. The *SCARNA3* gene also encodes a small nucleolar RNA similar in structure to *SNORA19*. The *RFWD12* gene encodes an E3 RING-type ubiquitin-protein ligase, which selects proteins for proteasomal degradation [34]. Many abnormal proteins are proteolyzed by the ubiquitin system as part of the stress response in eukaryotes [35]. Moreover, a total of 28 genes identified as being regulated by heat shock in the bovine embryo are associated with ubiquitin C [31].

The second region of interest on BTA 16 is located at about 35,272,426 bp. The closest genes to this area are centrosomal protein of 170 kDa (*CEP170*) and inactive phospholipase D5 (*PLD5*). Neither gene has an obvious relationship with physiological response to heat stress.

A region of BTA 5 at approximately 89,500,000 bp was consistently identified across analyses. Two genes flank the consensus region, solute carrier organic anion transporter family member 1C1 (*SLCO1C1*) and a phosphodiesterase (*PDE3A*). In humans, *SLCO1C1* mediates the Na^+^-independent high-affinity transport of thyroxine and reverse triiodothyronine [36]. Perhaps this gene is involved in the mechanism which depresses plasma thyroxine concentrations in heat-stressed dairy cows [37]. Human heat shock protein 70 has been shown to increase the enzymatic activity of phosphodiesterase in heat-shocked cells [38] and there may be a similar association in cattle.

The 4- and 5-SNP analyses showed a peak near 64,400,000 bp on BTA 4 that explained 0.09–0.11% of the observed variance (Tables S3 and 3). This region includes the genes kelch repeat and BTB domain-containing protein (*KBTBD2*) and U6 snRNA-associated Sm-like protein LSM5 (*LSM5*). *KBTBD2* encodes for a protein that participates in ubiquitination of proteins [39] while *LSM5* is likely to participate in RNA processing and to form part of the stress granule seen in stressed-cells that contains mRNAs stalled in translation [40].

A region centered on the SNP at 20,290,497 bp on BTA 26 explained 0.10% to 0.22% of the observed variance in the 2-SNP to 5-SNP analyses (Tables 2 and 3 and S1–S3. The SNP is located in the glutamine-oxaloacetic transaminase, soluble (*GOT1*) gene. *GOT1* is involved in synthesis of sulfur dioxide, which has been shown to reduce formation of reactive oxygen products and protect rat myocardial tissue in response to isoproterenol [41].

In an earlier study, Hayes et al. [42] identified a SNP on BTA 29 (BFGL-NGS-30169) that was associated with genetic variation in effects of heat stress on milk yield in Holsteins and Jerseys. A nearby SNP on BTA 29 (ARS-BFGL-NGS-107395 at 47,527,067 bp) was one of the top 30 markers in the 2- through 5-SNP analyses, ranking between the 19^th^ and 35^th^ largest SNP. It is located 355,843 bp upstream of the SNP identified by Hayes et al. [42] in a contig that includes only an annotated small nucleolar RNA (*SNORD14*).

There are small but significant genetic correlations of RT during heat stress with 305-d milk, fat, and protein yields, productive life, net merit, somatic cell score and daughter pregnancy rate [8]. Regardless of the analysis used to identify SNPs explanatory for RT, none of the 10 largest explanatory loci for RT were in common with 1,586 SNP markers related to 31 other traits in dairy cattle previously identified by Cole et al. [11]. Thus, it might be possible to use SNPs associated with RT to select for thermotolerance without inadvertent selection for other traits.

In conclusion, these data demonstrate that QTL exist to predict some of the genetic variation in RT. These QTL may prove important for genetic selection of thermotolerance. The challenge of performing GWAS for traits with low heritability when the number of phenotyped animals is low means that confirmation of the utility of these QTL is essential. In addition, several candidate genes for regulation of RT in dairy cattle were identified and one or more of these may play an important role in physiological adaptation to heat stress. Only one candidate gene, *SLC01C1*, which is involved in regulation of metabolic rate through transport of thyroxine, plays a known role in processes controlling body temperature. More commonly, candidate genes play roles that are important for stabilizing cellular function during stress. Among these are *GOT1*, which synthesizes the cytorotective compound sulphur dioxide, genes involved in protein ubiquitination (*KBTBD2 and RFWD12*), and genes involved in RNA metabolism (*LSM5, SCARNA3, SNORA19,* and *U1*).

## Supporting Information

Figure S1 Proportion of SNP variance explained by individual SNP effects for rectal temperature from a single-step GBLUP analysis.
(PNG)

Figure S2 Proportion of SNP variance explained by 2-SNP moving windows for rectal temperature from a single-step GBLUP analysis.
(PNG)

Figure S3 Proportion of SNP variance explained by 4-SNP moving windows for rectal temperature from a single-step GBLUP analysis.
(PNG)

Figure S4 Proportion of SNP variance explained by 10-SNP moving windows for rectal temperature from a single-step GBLUP analysis.
(PNG)

Table S1 The 20 loci with the largest single-SNP proportion of SNP variance explained for rectal temperature.
(PDF)

Table S2 The 20 loci with the largest proportion of SNP variance explained for rectal temperature using 2-SNP sliding windows.
(PDF)

Table S3 The 20 loci with the largest proportion of SNP variance explained for rectal temperature using 4-SNP sliding windows.
(PDF)

Table S4 The 20 loci with the largest proportion of SNP variance explained for rectal temperature using 10-SNP sliding windows.
(PDF)

File S1 The name, chromosome number, SNP location (BP), and proportion of SNP variance for rectal temperature explained by each SNP from a single-step GBLUP analysis in CSV format. Marker locations are based on the UMD 3.1 assembly of the bovine genome.
(CSV)

## References

[1] West JW (2003) Effects of heat-stress on production in dairy cattle. J Dairy Sci 86: 2131–2144. 12836950 10.3168/jds.S0022-0302(03)73803-X

[2] Hansen PJ (2007) Exploitation of genetic and physiological determinants of embryonic resistance to elevated temperature to improve embryonic survival in dairy cattle during heat stress. Theriogenology 68S: 242–249. 10.1016/j.theriogenology.2007.04.008 17482669

[3] Elvinger F, Natzke RP, Hansen PJ (1992) Interactions of heat stress and bovine somatotropin affecting physiology and immunology of lactating cows. J Dairy Sci 75: 449–462. 1560140 10.3168/jds.S0022-0302(92)77781-9

[4] Hansen PJ (2011) Heat stress and climate change. In: Moo-Young M, (ed.), *Comprehensive Biotechnology*, Second Edition, vol. 4, 477–485. Amsterdam: Elsevier.

[5] Liu Y, Dagi L, Li H, Zhou X (2011) A novel SNP of the ATP1A1 gene is associated with heat tolerance in dairy cows. Mol Biol Rep 38: 83–88. 20336380 10.1007/s11033-010-0080-8

[6] Olson TA, Lucena C, Chase CC Jr, Hammond AC (2003) Evidence of a major gene influencing hair length and heat tolerance in *Bos taurus* cattle. J Anim Sci 81: 80–90. 12597376 10.2527/2003.81180x

[7] Seath DM (1947) Heritability of heat tolerance in dairy cattle. J. Dairy Sci. 30: 137–144.

[8] Dikmen S, Cole JB, Null DJ, Hansen PJ (2012) Heritability of rectal temperature and genetic correlations with production and reproduction traits in dairy cattle. J Dairy Sci 95: 3401–3405. 22612974 10.3168/jds.2011-4306

[9] VanRaden PM, Van Tassell CP, Wiggans GR, Sonstegard TS, Schnabel RD, et al (2009) Invited review: Reliability of genomic predictions for North American Holstein bulls. J Dairy Sci 92: 16–24. 19109259 10.3168/jds.2008-1514

[10] Hayes BJ, Pryce J, Chamberlain AJ, Bowman PJ, Goddard ME (2010) Genetic architecture of complex traits and accuracy of genomic prediction: coat colour, milk-fat percentage, and type in Holstein cattle as contrasting model traits. PLoS Genet 6: e1001139. 20927186 10.1371/journal.pgen.1001139 PMC2944788

[11] Cole JB, Wiggans GR, Ma L, Sonstegard TS, Lawlor TJ Jr, et al (2011) Genome-wide association analysis of thirty one production, health, reproduction and body conformation traits in contemporary U.S. Holstein cows. BMC Genomics 12: 408–465. 21831322 10.1186/1471-2164-12-408 PMC3176260

[12] Wiggans GR, Vanraden PM, Cooper TA (2011) The genomic evaluation system in the United States: past, present, future. J Dairy Sci 94: 3202–3211. 21605789 10.3168/jds.2010-3866

[13] Berry DP, Bastiaansen JW, Veerkamp RF, Wijga S, Wall E, et al (2012) Genome-wide associations for fertility traits in Holstein-Friesian dairy cows using data from experimental research herds in four European countries. Animal 6: 1206–1215. 23217223 10.1017/S1751731112000067

[14] Pfahler S, Distl O (2012) Identification of quantitative trait loci (QTL) for canine hip dysplasia and canine elbow dysplasia in Bernese mountain dogs. PLoS One 7: e49782. 23189162 10.1371/journal.pone.0049782 PMC3506637

[15] National Research Council (1971) *A Guide to Environmental Research on Animals.* Natl. Acad. Sci., Washington, DC.

[16] Dikmen S, Hansen PJ (2009) Is the temperature-humidity index the best indicator of heat stress in lactating dairy cows in a subtropical environment? J Dairy Sci 92: 109–116. 19109269 10.3168/jds.2008-1370

[17] Thompson R, Mantysaari EA (1999) Prospects for statistical methods in dairy cattle breeding. Interbull Bull 20: 70–79.

[18] Misztal I, Tsuruta S, Strabel T, Auvray B, Druet T, et al.. (2002) BLUPF90 and related programs (BGF90). Proc 7th World Congr Genet Appl Livest Prod Commun No 28–07.

[19] Wang H, Misztal I, Aguillar I, Legarra A, Muir WM (2012) Genome­wide association mapping including phenotypes from relatives without genotypes. Genet Res 94: 73–83. 10.1017/S0016672312000274 22624567

[20] Legarra A, Aguilar I, Misztal I (2009) A relationship matrix including full pedigree and genomic information. J Dairy Sci 92: 4656–4663. 19700729 10.3168/jds.2009-2061

[21] Misztal I, Legarra A, Aguilar I (2009) Computing procedures for genetic evaluation including phenotypic, full pedigree, and genomic information. J Dairy Sci 92: 4648–4655. 19700728 10.3168/jds.2009-2064

[22] Aguilar I, Misztal I, Johnson DL, Legarra A, Tsuruta S, et al (2010) Hot topic: A unified approach to utilize phenotypic, full pedigree, and genomic information for genetic evaluation of Holstein final score. J Dairy Sci 93: 743–752. 20105546 10.3168/jds.2009-2730

[23] Wickham H (2009) *ggplot2: Elegant Graphics for Data Analysis*. Springer, New York, NY, USA.

[24] R Development Core Team (2010) *R: A Language and Environment for Statistical Computing.* R Foundation for Statistical Computing, Vienna, Austria.

[25] Zondervan KT, Cardon LR (2004) The complex interplay among factors that influence allelic association. Nature Rev Genetics 5: 89–100. 14735120 10.1038/nrg1270

[26] Servin B, Stephens M (2007) Imputation-based analysis of association studies: candidate regions and quantitative traits. PLoS Genet 3: e114. 17676998 10.1371/journal.pgen.0030114 PMC1934390

[27] Ioannidis JPA (2013) This I believe in genetics: discovery can be a nuisance, replication is science, implementation matters. Front Genet 4: 33. 23505393 10.3389/fgene.2013.00033 PMC3596761

[28] Sargolzaei M, Schenkel FS, Jansen GB, Schaeffer LR (2008) Extent of linkage disequilibrium in Holstein cattle in North America. J Dairy Sci 91: 2106–2117. 18420642 10.3168/jds.2007-0553

[29] Berg MG, Singh LN, Younis I, Liu Q, Pinto AM, et al (2012) U1 snRNP determines mRNA length and regulates isoform expression. Cell 150: 53–64. 22770214 10.1016/j.cell.2012.05.029 PMC3412174

[30] Collier RJ, Collier JL, Rhoads RP, Baumgard LH (2008) Invited Review: Genes involved in the bovine heat stress response. J Dairy Sci 91: 445–454. 18218730 10.3168/jds.2007-0540

[31] Sakatani M, Bonilla L, Dobbs KB, Block J, Ozawa M, et al (2013) Changes in the transcriptome of morula-stage bovine embryos caused by heat shock: relationship to developmental acquisition of thermotolerance. Reprod Biol Endocrinol 11: 3. 23320502 10.1186/1477-7827-11-3 PMC3583805

[32] Aho AD, McNulty AM, Coussens PM (2003) Enhanced expression of interleukin-1α and tumor necrosis factor receptor-associated protein 1 in ileal tissues of cattle infected with *Mycobacterium avium* subsp. *paratuberculosis* . Infect Immun 71: 6479–6486. 14573670 10.1128/IAI.71.11.6479-6486.2003 PMC219597

[33] Tollervey D, Kiss T (1997) Function and synthesis of small nucleolar RNAs. Curr Opin Cell Biol 9: 337–342. 9159079 10.1016/s0955-0674(97)80005-1

[34] Robinson PA, Ardley HC (2004) Ubiquitin-protein ligases. J Cell Sci 117: 5191–5194. 15483315 10.1242/jcs.01539

[35] Parsell DA, Lindquist S (1993) The function of heat -shock proteins in stress tolerance: Degradation and reactivation of damaged proteins. Ann Rev Genet 27: 437–496. 8122909 10.1146/annurev.ge.27.120193.002253

[36] Pizzagalli F, Hagenbuch B, Stieger B, Klenk U, Folkers G, et al (2002) Identification of a novel human organic anion transporting polypeptide as a high affinity thyroxine transporter Mol Endocrinol. 16: 2283–2296. 10.1210/me.2001-0309 12351693

[37] Scott IM, Johnson HD, Hahn GL (1983) Effect of programmed diurnal temperature cycles on plasma thyroxine level, body temperature, and feed intake of Holstein dairy cows. Int J Biometeorol 27: 47–62. 6683712 10.1007/BF02186300

[38] Kiang JG, Tsokos GC (1998) Heat shock protein 70 kDa: Molecular biology, biochemistry, and physiology. Pharmacol Therapeut 80: 183–201. 10.1016/s0163-7258(98)00028-x 9839771

[39] Marshall J, Blair LA, Singer JD (2011) BTB-Kelch proteins and ubiquitination of kainate receptors. Adv Exp Med Biol 717: 115–125. 21713671 10.1007/978-1-4419-9557-5_10 PMC3929045

[40] Balagopal V, Parker R (2009) Polysomes, P bodies and stress granules: states and fates of eukaryotic mRNAs. Curr Opin Cell Biol 21: 403–408. 19394210 10.1016/j.ceb.2009.03.005 PMC2740377

[41] Liang Y, Liu D, Ochs T, Tang C, Chen S, et al (2011) Endogenous sulfur dioxide protects against isoproterenol-induced myocardial injury and increases myocardial antioxidant capacity in rats. Lab Invest 91: 12–23. 20733562 10.1038/labinvest.2010.156

[42] Hayes BJ, Bowman PJ, Chamberlain AJ, Savin K, van Tassell CP, et al (2009) A validated genome wide association study to breed cattle adapted to an environment altered by climate change. PLoS One 4: e6676. 19688089 10.1371/journal.pone.0006676 PMC2722733

