# Supplementary figures and images for: Genome-Wide Association Mapping for Identification of Quantitative Trait Loci for Rectal Temperature during Heat Stress in Holstein Cattle
Source: PLoS One. 2013 Jul 23;8(7):e69202. doi: 10.1371/journal.pone.0069202 (PMC3720646; doi:10.1371/journal.pone.0069202)

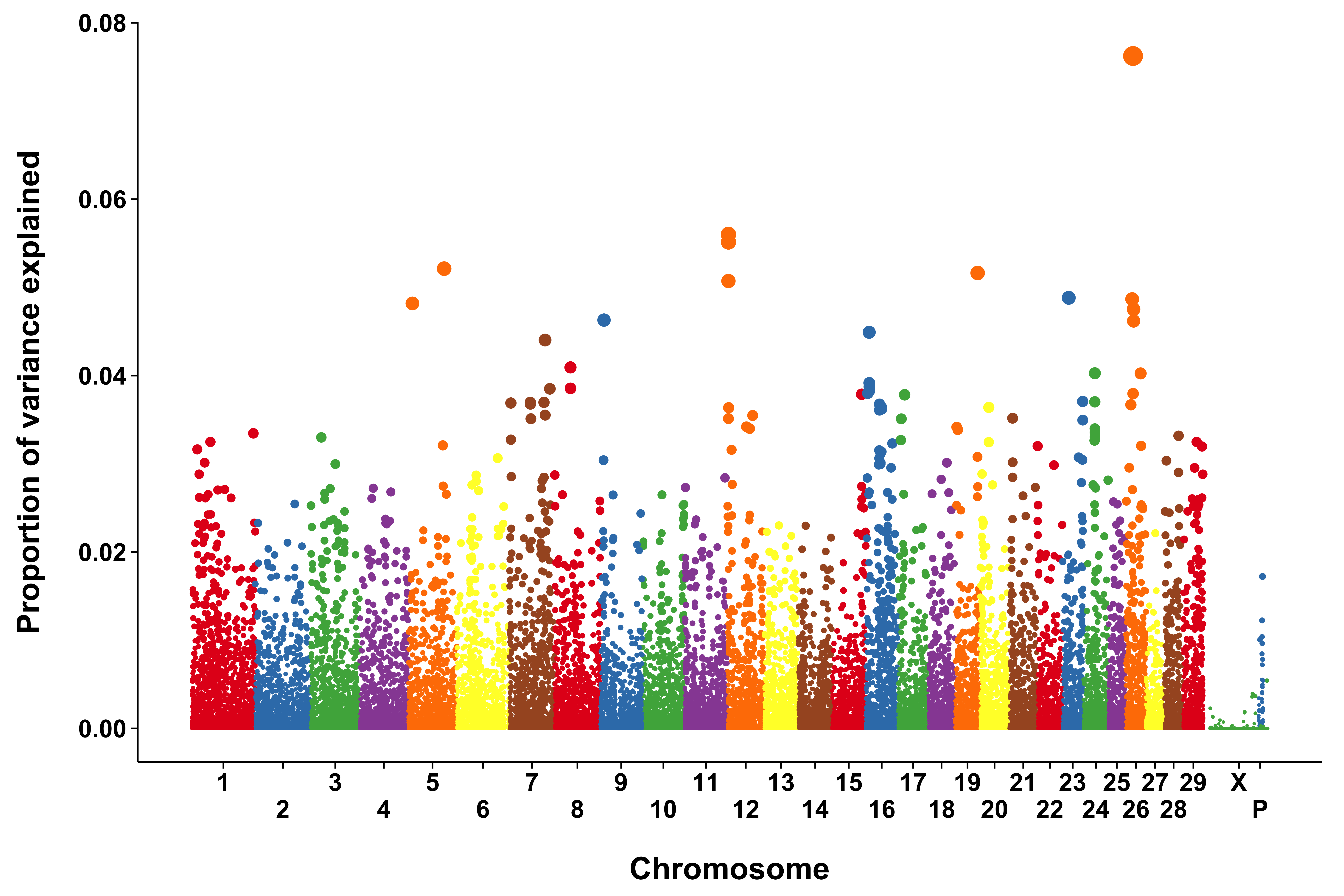

Supplement: Figure S1 — Proportion of SNP variance explained by individual SNP effects for rectal temperature from a single-step GBLUP analysis. (PNG) [file pone.0069202.s001.png]

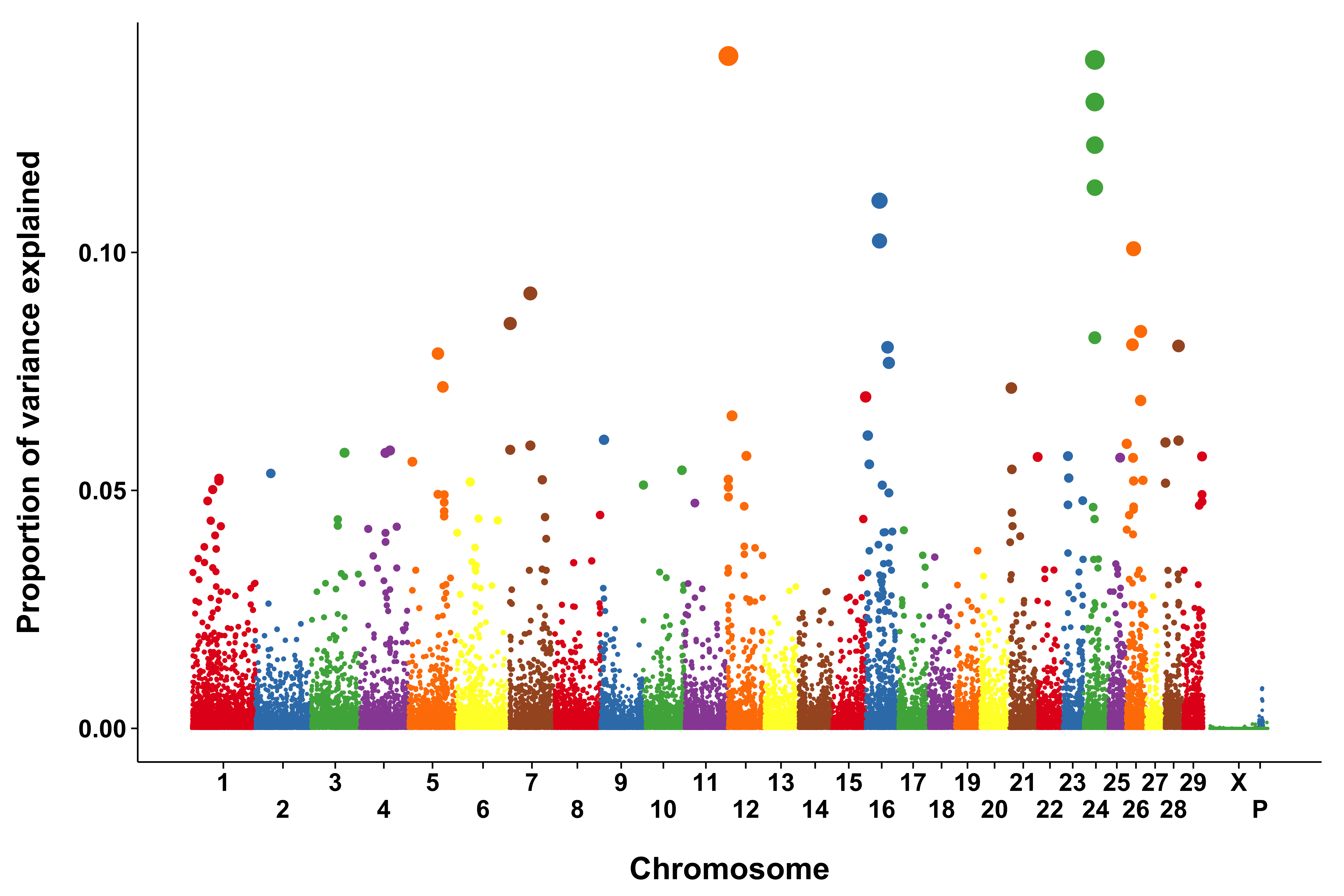

Supplement: Figure S2 — Proportion of SNP variance explained by 2-SNP moving windows for rectal temperature from a single-step GBLUP analysis. (PNG) [file pone.0069202.s002.png]

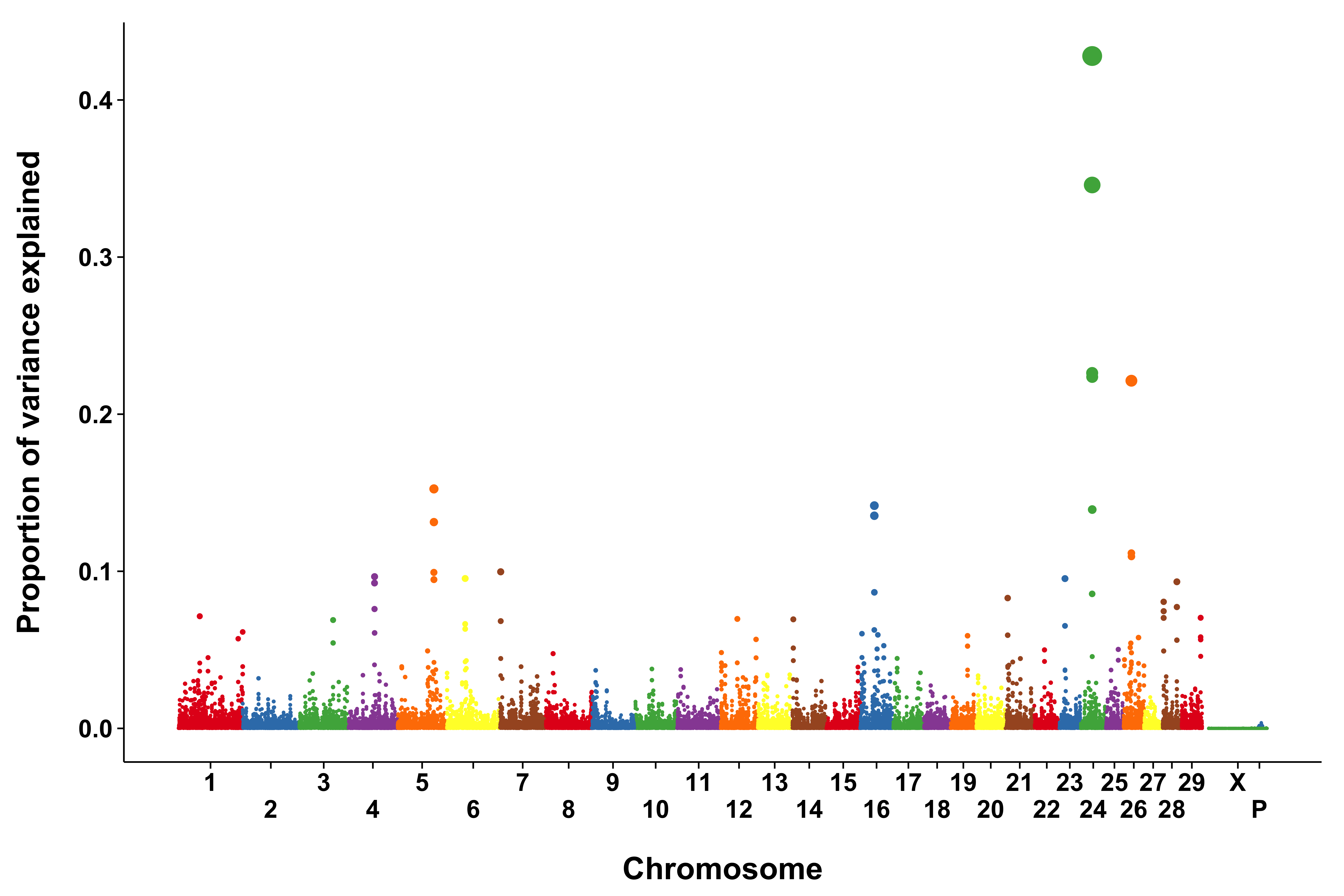

Supplement: Figure S3 — Proportion of SNP variance explained by 4-SNP moving windows for rectal temperature from a single-step GBLUP analysis. (PNG) [file pone.0069202.s003.png]

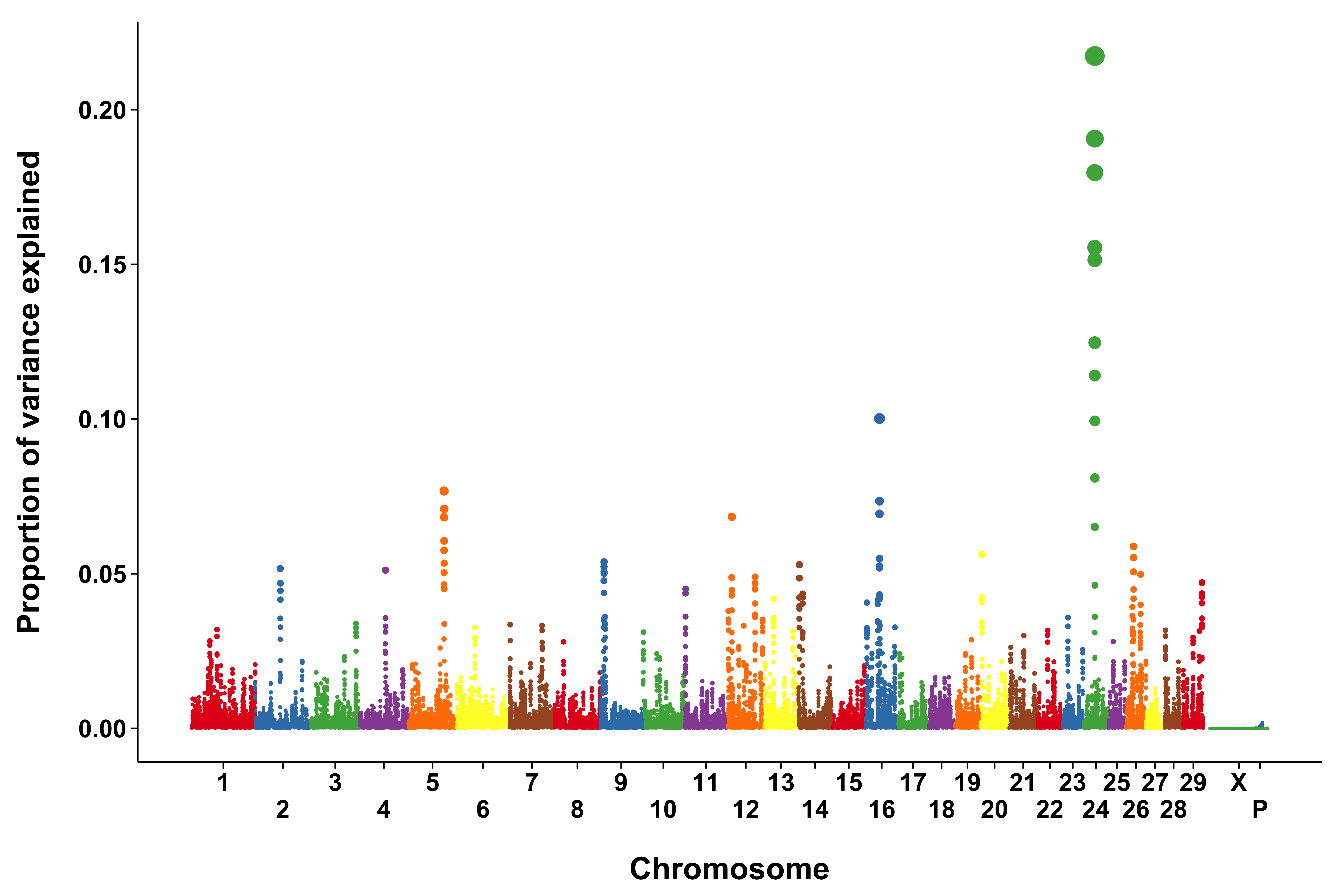

Supplement: Figure S4 — Proportion of SNP variance explained by 10-SNP moving windows for rectal temperature from a single-step GBLUP analysis. (PNG) [file pone.0069202.s004.png]
