# Supplementary material for: Genome-Wide Association Mapping for Identification of Quantitative Trait Loci for Rectal Temperature during Heat Stress in Holstein Cattle
Source: PLoS One. 2013 Jul 23;8(7):e69202. doi: 10.1371/journal.pone.0069202 (PMC3720646; doi:10.1371/journal.pone.0069202)
Supplement: Table S1 — The 20 loci with the largest single-SNP proportion of SNP variance explained for rectal temperature. (PDF) [file pone.0069202.s005.pdf]

Table S1. The 20 loci with the largest single-SNP proportion of SNP variance explained for rectal temperature.

SNP name	Chromosome	Location (bp)	Variance explained (%)
ARS-BFGL-NGS-97471	26	18882047	0.08
BTA-27507-no-rs	12	2618039	0.06
Hapmap49942-BTA-27508	12	2682385	0.06
Hapmap47861-BTA-120563	5	89472174	0.05
ARS-BFGL-NGS-28678	19	57454104	0.05
BTB-01103269	12	2569573	0.05
Hapmap8049-BTA-55656	23	16171880	0.05
Hapmap41595-BTA-60800	26	16791783	0.05
BTA-119598-no-rs	5	10626452	0.05
ARS-BFGL-NGS-71584	26	20290497	0.05
Hapmap41153-BTA-110528	9	10134275	0.05
ARS-BFGL-NGS-2464	26	20444634	0.05
BTB-01221760	16	9640741	0.04
ARS-BFGL-NGS-116915	7	89026917	0.04
Hapmap48952-BTA-94977	8	39745401	0.04
BTB-01646599	24	28941584	0.04
ARS-BFGL-NGS-95833	26	37797893	0.04
ARS-USMARC-Parent-DQ846693-rs29017621	16	9855276	0.04
Hapmap58503-rs29017623	16	10061745	0.04
Hapmap55166-rs29016846	8	39836688	0.04
