# Supplementary material for: Genome-Wide Association Mapping for Identification of Quantitative Trait Loci for Rectal Temperature during Heat Stress in Holstein Cattle
Source: PLoS One. 2013 Jul 23;8(7):e69202. doi: 10.1371/journal.pone.0069202 (PMC3720646; doi:10.1371/journal.pone.0069202)
Supplement: Table S2 — The 20 loci with the largest proportion of SNP variance explained for rectal temperature using 2-SNP sliding windows. (PDF) [file pone.0069202.s006.pdf]

Table S2. The 20 loci with the largest proportion of SNP variance explained for rectal temperature using 2-SNP sliding windows.

SNP name	Chromosome	Location (bp)	Variance explained (%)
BTB-01103269	12	2569573	0.14
BTB-01646599	24	28941584	0.14
Hapmap58887-rs29013502	24	28907154	0.13
ARS-BFGL-NGS-41140	24	28975828	0.12
ARS-BFGL-NGS-35716	24	29013292	0.11
Hapmap46698-BTA-38760	16	35317388	0.11
BTB-00638221	16	35272426	0.10
ARS-BFGL-NGS-71584	26	20290497	0.10
ARS-BFGL-NGS-33637	7	52482580	0.09
BTB-00291825	7	2486342	0.09
ARS-BFGL-NGS-107207	26	37871121	0.08
BTB-01485274	24	28877547	0.08
BTA-62092-no-rs	26	17437785	0.08
UA-IFASA-5633	28	35354394	0.08
Hapmap39827-BTA-39283	16	55209290	0.08
ARS-BFGL-NGS-113371	5	74087300	0.08
ARS-BFGL-NGS-108847	16	58500249	0.08
Hapmap51489-BTA-74505	5	86232490	0.07
ARS-BFGL-NGS-44523	21	6035370	0.07
ARS-BFGL-NGS-24582	15	85049720	0.07
