# Supplementary material for: Genome-Wide Association Mapping for Identification of Quantitative Trait Loci for Rectal Temperature during Heat Stress in Holstein Cattle
Source: PLoS One. 2013 Jul 23;8(7):e69202. doi: 10.1371/journal.pone.0069202 (PMC3720646; doi:10.1371/journal.pone.0069202)
Supplement: Table S3 — The 20 loci with the largest proportion of SNP variance explained for rectal temperature using 4-SNP sliding windows. (PDF) [file pone.0069202.s007.pdf]

Table S3. The 20 loci with the largest proportion of SNP variance explained for rectal temperature using 4-SNP sliding windows.

SNP name	Chromosome	Location (bp)	Variance explained (%)
BTB-01646599	24	28941584	0.43
Hapmap58887-rs29013502	24	28907154	0.35
BTB-01485274	24	28877547	0.23
ARS-BFGL-NGS-41140	24	28975828	0.22
ARS-BFGL-NGS-71584	26	20290497	0.22
BTB-01267098	5	89545151	0.15
BTB-00638221	16	35272426	0.14
ARS-BFGL-NGS-35716	24	29013292	0.14
ARS-BFGL-NGS-100932	16	35230105	0.14
BTB-01267080	5	89512928	0.13
ARS-BFGL-NGS-10307	26	20259486	0.11
ARS-BFGL-NGS-23064	26	20365711	0.11
BTB-02004898	7	2391922	0.10
Hapmap47861-BTA-120563	5	89472174	0.10
ARS-BFGL-NGS-68143	4	64492908	0.10
Hapmap30420-BTC-039335	6	45175137	0.10
ARS-BFGL-NGS-100006	23	14215024	0.10
BTB-01267042	5	89568937	0.10
BTA-26221-no-rs	28	35345760	0.09
Hapmap39941-BTA-70878	4	64386271	0.09
