# Supplementary material for: Genome-Wide Association Mapping for Identification of Quantitative Trait Loci for Rectal Temperature during Heat Stress in Holstein Cattle
Source: PLoS One. 2013 Jul 23;8(7):e69202. doi: 10.1371/journal.pone.0069202 (PMC3720646; doi:10.1371/journal.pone.0069202)
Supplement: Table S4 — The 20 loci with the largest proportion of SNP variance explained for rectal temperature using 10-SNP sliding windows. (PDF) [file pone.0069202.s008.pdf]

Table S4. The 20 loci with the largest proportion of SNP variance explained for rectal temperature using 10-SNP sliding windows.

SNP name	Chromosome	Location (bp)	Variance explained (%)
BTB-01485274	24	28877547	0.22
Hapmap58887-rs29013502	24	28907154	0.19
Hapmap54981-rs29019846	24	28853585	0.18
BTB-01646599	24	28941584	0.16
ARS-BFGL-BAC-28665	24	28824671	0.15
BTA-97262-no-rs	24	28770460	0.12
ARS-BFGL-NGS-41140	24	28975828	0.11
BTB-00638221	16	35272426	0.10
ARS-BFGL-NGS-5141	24	28772509	0.10
ARS-BFGL-NGS-35716	24	29013292	0.08
BTB-01267305	5	89425557	0.08
Hapmap46698-BTA-38760	16	35317388	0.07
BTB-01267098	5	89545151	0.07
ARS-BFGL-NGS-100932	16	35230105	0.07
ARS-BFGL-NGS-43211	12	11179949	0.07
BTB-01267080	5	89512928	0.07
BTB-01485885	24	28691752	0.07
Hapmap47861-BTA-120563	5	89472174	0.06
ARS-BFGL-NGS-23064	26	20365711	0.06
BTB-01790876	5	89376970	0.06
